# Sertoli Cell-Only Syndrome: Behind the Genetic Scenes

**DOI:** 10.1155/2016/6191307

**Published:** 2016-01-26

**Authors:** Katrien Stouffs, Alexander Gheldof, Herman Tournaye, Deborah Vandermaelen, Maryse Bonduelle, Willy Lissens, Sara Seneca

**Affiliations:** ^1^Center for Medical Genetics/Research Center Reproduction and Genetics, Universitair Ziekenhuis Brussel, Vrije Universiteit Brussel, Laarbeeklaan 101, 1090 Brussels, Belgium; ^2^Center for Reproductive Medicine/Biology of the Testis, Universitair Ziekenhuis Brussel, Vrije Universiteit Brussel, Laarbeeklaan 101, 1090 Brussels, Belgium

## Abstract

Sertoli cell-only syndrome is defined by the complete absence of germ cells in testicular tissues and always results in male infertility. The aetiology often remains unknown. In this paper, we have investigated possible causes of Sertoli cell-only syndrome with a special focus on genetic causes. Our results show that, for a large part of the patients (>23% in an unselected group), the sex chromosomes are involved. The majority of patients had a Klinefelter syndrome, followed by patients with Yq microdeletions. Array comparative genomic hybridization in a selected group of “idiopathic patients” showed no known infertility related copy number variations.

## 1. Introduction

Spermatogenesis is a very complex process, involving thousands of genes, which can have an ubiquitous expression pattern or can have a specific function in reproductive tissues or spermatogenesis [1, 2]. Furthermore, many alternatively spliced spermatogenesis-specific transcripts are only detected in the testis, pointing at specialized functions of genes with an otherwise general expression pattern. The development of computation programs (fi the NCBI tool ORF finder: http://www.ncbi.nlm.nih.gov/gorf/gorf.html) has facilitated the prediction of presumable protein-coding genes. Moreover, massive parallel sequencing technologies have greatly facilitated the identification of transcripts and genes involved in spermatogenesis and other tissues. However, the characterization of the functionality and importance of newly identified genes remain a challenge.

In this study, we focus on a testicular phenotype in which all spermatogenic cells are missing: Sertoli cell-only syndrome. Patients with Sertoli cell-only syndrome are infertile due to nonobstructive azoospermia (NOA). Although, for most of these patients, artificial reproductive techniques such as intracytoplasmic sperm injection (ICSI) with the patients' own sperm cells will be impossible, it is important to gain insight into the origin of the fertility problems. For the small number of patients for whom still a few spermatozoa could be obtained, often after testicular sperm extractions (TESE), it is even more essential to gain insight into potential genetic origins of their problems. For these patients ICSI might be possible and, consequently, the fertility problems might be transmitted to the next generation.

In this study, we have examined patients with Sertoli cell-only syndrome. We first studied known (genetic) causes of Sertoli cell-only syndrome and then looked at the presence of copy number variations by array comparative genomic hybridization (array CGH) analysis in a selected group of patients with “idiopathic” Sertoli cell-only syndrome.

## 2. Material and Methods

### 2.1. Selection Criteria for Patients and Controls

**Figure 1:**
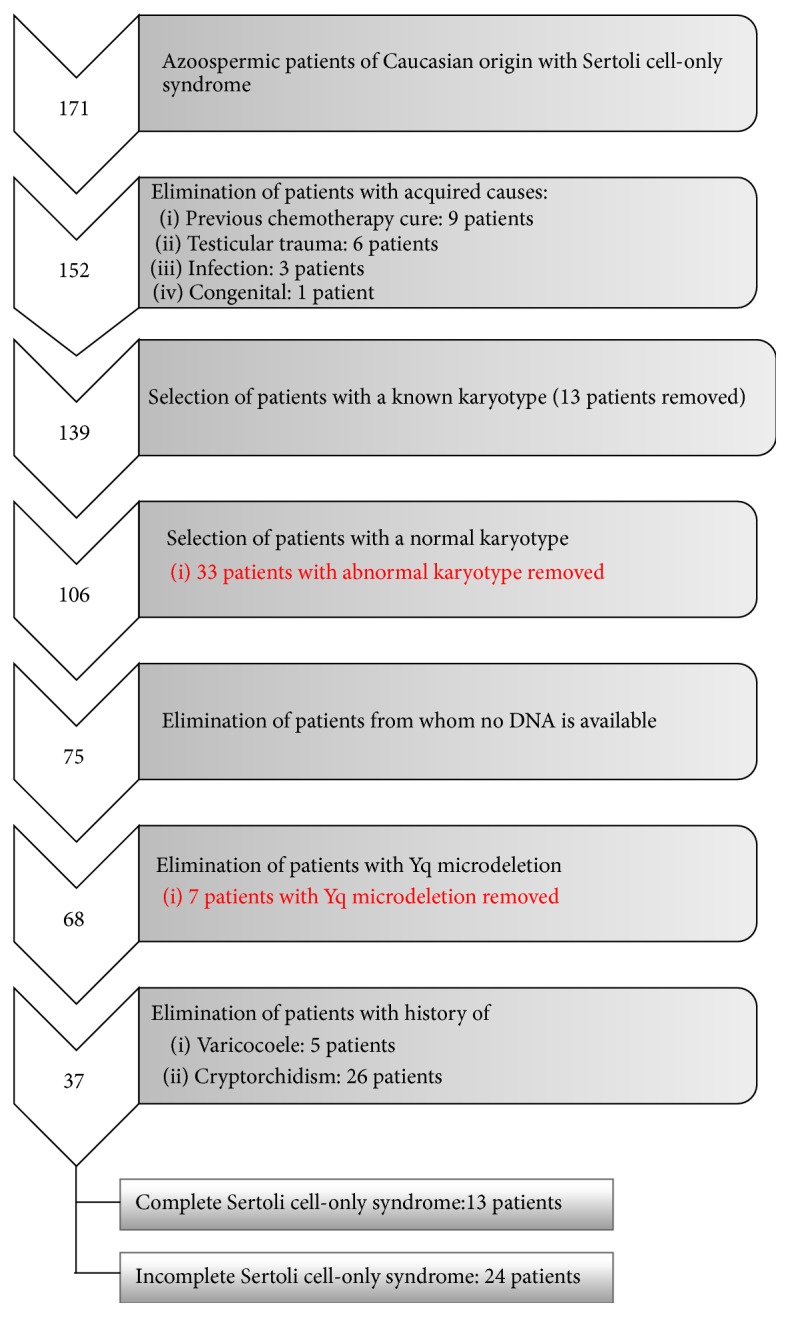
Overview of the patient selection.

The selection and elimination criteria for the patients are shown in Figure 1. A total of 171 azoospermic Caucasian patients with Sertoli cell-only syndrome were included in the present study. The diagnosis of azoospermia was based on at least two routine semen analyses, while a further classification as Sertoli cell-only syndrome was based on the histological examination of a testicular biopsy within the frame of their fertility work-up [3].

For array CGH analysis, a selected group of patients was analyzed. The exclusion criteria included abnormal karyotype, presence of Yq microdeletion, presence of gr/gr deletion, previous varicocoele, or history of cryptorchidism. Furthermore, for all patients TESE was performed, and patients with residual spermatogenesis were excluded, defined as “incomplete Sertoli cell-only syndrome.”

For the control group, DNA samples from men with normozoospermia, defined by routine sperm analysis, were used. These men were also from Caucasian origin from Belgium or The Netherlands. For array CGH analysis, 23 control samples were included, but these numbers are increased for the subanalyses as mentioned in the text.

### 2.2. DNA Preparation

Genomic DNA was isolated from peripheral blood using magnetic purification with the “Multiprobe II Plus EX + Gripper” liquid handling robot and “Chemagic Magnetic Separation Module I” (PerkinElmer, Belgium).

### 2.3. Yq Microdeletion Analysis and Karyotype Analysis

Karyotype analyses were performed using routine analyses. Yq microdeletion analysis was performed according to the guidelines proposed by Simoni et al. [4].

### 2.4. Array CGH

Array CGH analysis was performed using standard methods described [5]. In brief, 300 ng of genomic DNA was labeled with Cy3-dCTP or Cy5-dCTP (GE Healthcare, Belgium) using Bioprime array CGH genomic labeling system (Invitrogen, Belgium). For the labeling, we used the “triangle method”: DNA samples from patients and controls were labeled and hybridized using a dye swap in trios consisting of at least one control per triangle. Samples were hybridized on 244K arrays (design ID 014693, Agilent, Belgium) for 40 h at 65°C. After washing, the samples were scanned at 5 *μ*m resolution using a DNA microarray scanner G2505B (Agilent, Belgium). The scan images were analyzed using the feature extraction software 9.5.3.1 (Agilent) and further analyzed with “arrayCGHbase” [6]. Copy number variations were taken into consideration when two or more flanking probes were exceeding a value of the intensity ratios ± four times the standard deviation of log_2_ of all intensity ratios for that experiment. Always two experiments investigating the same sample with a dye swap were compared and only when an alteration is present in both experiments was the region included for further analysis. Inconsistencies were inspected manually.

### 2.5. qPCR

qPCR was performed on genomic DNA using predesigned Taqman Copy Number Assays (Applied Biosystems, Belgium) according to instructions of the manufacturer. Samples were run on the 7500 Real Time PCR system (Applied Biosystems, Belgium) and analyzed using CopyCaller Software provided by Applied Biosystems (Belgium). The assays used are reported in Supplementary Table 1 in Supplementary Material available online at http://dx.doi.org/10.1155/2016/6191307. In each assay, we have analyzed the patient with the alteration detected by array CGH.

### 2.6. RNA Expression

The presence of RNA in testicular tissues was investigated using home-made RNA. Fresh testicular tissue was obtained from patients who came to the hospital for vasectomy repair and who signed an informed consent. The histology was determined on a second biopsy and showed normal spermatogenesis. RNA was extracted using the RNeasy Mini kit (Qiagen, Belgium) after which cDNA was prepared using the Transcriptor First Strand cDNA Synthesis Kit (Roche, Belgium). Primers for amplification of cDNA were designed according to the reference sequences and were overlapping at least one intron/exon boundary (primers available upon request). All amplified fragments from testicular tissues were sequenced to confirm specific amplification. Commercially available “Human Universal Reference Total RNA” (Westburg, Belgium) was investigated according to the above described method. The expression of RNA in multiple human tissues was analyzed using the Human MTC panels I and II including cDNA from 16 different tissues (Clontech, Westburg, Belgium). For MTHFD2L, two fragments were obtained after amplification with forward primer TTGTGCCTTGATCAGCATTC and reverse primer TGTCACTGGATCGTGGACAT, located in exon 4 and exon 7, respectively. The two obtained fragments were purified from an agarose gel after electrophoresis using the QIAEXII Gel Extraction kit (Qiagen) according to instructions of the manufacturer. The fragments were reamplified and sequenced using the above mentioned primers.

### 2.7. ncRNA Analysis

The analysis of “geneless” regions for the presence of noncoding (nc)RNA or presumed transcripts was performed using the following online programs: http://genome.ucsc.edu/, http://www.ensembl.org/index.html, http://diana.imis.athena-innovation.gr/DianaTools/index.php (miRGen2.0 was used) [7], and http://www.mirbase.org/search.shtml [8].

## 3. Results

### 3.1. Patient Selection

From 1994 to 2007, more than 600 patients with nonobstructive azoospermia have consulted the Center for Reproductive Medicine for a fertility treatment [9]. From this patient group, we have selected 171 Caucasian men that were categorized as having Sertoli cell-only syndrome. From this group, patients with idiopathic Sertoli cell-only syndrome were selected (Figure 1).

We were, in a first step, interested in known genetic causes of Sertoli cell-only syndrome. Therefore, we looked at the karyotype of the patients. For 139 patients, a karyotype was available (performed either in our lab or elsewhere). In this group 33 patients had an abnormal karyotype (24%). The majority of these individuals were Klinefelter patients (*n* = 29) or mosaics 46,XY/47,XXY (*n* = 3). The karyotype of the remaining patient was 46,X,der(X)t(Xp;Yp).

Next, we focused on Yq microdeletions. For the patients for whom DNA was available, we detected seven patients with a Yq microdeletion: four patients with an AZFc deletion, one patient with an AZFb deletion, and two patients with an AZFb + AZFc deletion.

Finally, we looked at potential risk factors for male infertility: a history of cryptorchidism or a (previous) varicocoele. These two conditions are known to potentially influence the fertility status of the patient, although a clear relationship remains controversial since, for some patients, sperm production is normal or only slightly subnormal.

For the remainder of the study, we ended up with 37 patients for whom the etiology of their fertility problems remains unknown. Thirteen of these patients had a complete Sertoli cell-only syndrome and for 24 patients some sperm cells could be retrieved during TESE. We focused on nine patients with a complete Sertoli cell-only syndrome. The four remaining patients were no longer available for further research.

### 3.2. Array CGH Analysis

In order to detect copy number variations, array CGH analysis was performed on the nine patient samples described above. Together with these patients, we have also analyzed 23 control samples.

**Figure 2:**
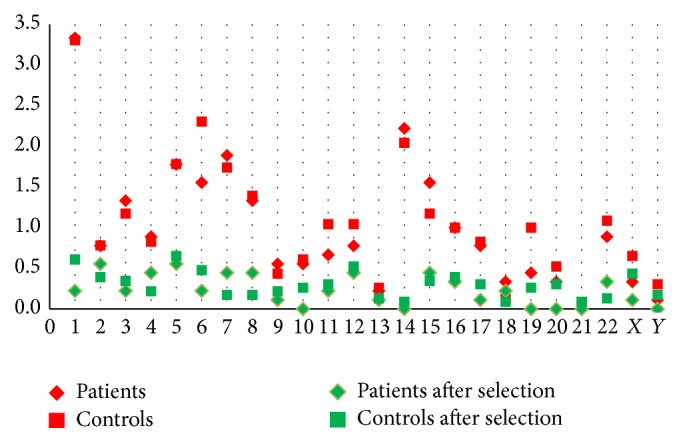
The average number of copy number variations per chromosome (CNVs) detected in the patient and control group before and after the removal of recurrent CNVs (i.e., CNVs that are detected in patients as well as in controls). The results do not differ between the two groups.

In total, we have detected 800 CNVs, 213 in the patient group and 587 in the control group. The average number of CNVs was not different in the patient versus the control group: 23.7 versus 25.5. Next, we looked at the number of CNVs for each chromosome (Figure 2, red spots). From the 800 regions, 587 regions (163 in the patient group and 424 in the control group) could be selected out because they were detected in patients as well as in controls. Figure 2 shows the average number of regions remaining after this selection procedure (green spots). In general, considering all chromosomes and after this selection, on average 5.6 CNVs were detected per patient, while this number was 7.1 in the control group. Finally, regions that were not containing any genes were eliminated. We ended up with 30 unique regions which were only altered in the patient group (Table 1). We also focused on “geneless regions” that are uniquely detected in the patient group and for which the reported CNV frequency is low (<5%). A total of 12 regions were analyzed through UCSC, Ensembl, the Diana database, and miRbase (Table 2).

### 3.3. Selection of Regions for qPCR Analysis to Look for CNVs in Patients and Controls

From the 30 unique gene-containing regions, the most promising CNVs were selected based on the reported population frequency and by searching the literature for the expression pattern, potential function of genes, and evolutionary conservation. Overall, 5 regions remained for further analysis (regions in bold in Table 1). These regions were three deletions located in chromosomal regions 4q13.3, 16p13.11, and 18q21.2 and two duplications located in 1q23.1 and 13q12.11. These regions varied in size from ~26 kb to ~147 kb. For all five regions, at least 87 extra normozoospermic controls were tested using Taqman copy number assays; together with the 23 normozoospermic controls tested through array CGH, a total of 110 controls were studied. In the analyses, a positive control, that is, the patient in which the CNV was detected, was always included. Consequently, all observed CNVs could be confirmed.

Multiple deletions have been detected for the region 16p13.11. We have tested a qPCR assay that was located in the PDXDC1 gene. This region was considered as probably nonpathogenic and therefore was not studied in more depth. For all remaining regions 40 more controls were examined with qPCR. In these groups, no deletions/duplications were detected, except for the positive control.

### 3.4. Expression Analysis of Candidate Infertility Genes

We have tested the expression pattern of the following genes, located in the CNV regions of interest (Table 1) using multiple tissue cDNA panels or testis derived cDNA (see Section 2): C18ORF26, MTHFD2L, PDXDC1, and PRCC. The genes PDXDC1 and PRCC showed a ubiquitous expression pattern. No mRNA for C18ORF26 could be detected, which is in accordance with published data reporting that C18ORF26 is barely expressed in (normal) human tissues [10].

We could detect MTHFD2L when analyzing total human RNA. By amplifying a fragment compromising exon 4 to exon 7, two PCR fragments were detected. Excision of these fragments from a gel followed by reamplification and sequencing of each fragment individually showed two alternatively spliced fragments. The longest fragment showed an insertion of 98 bp, located in intron 6 bases c.806-20313 to c.806-20216 (minus strand) according to RefSeq NM_001144978.1. On the other hand, no transcripts were detected in the testis.

## 4. Discussion

In this study, we investigated the frequency of genetic causes of Sertoli cell-only syndrome and tried to identify new genetic causes. It is obvious from this study that karyotype abnormalities (24%), especially Klinefelter syndrome, are the most common abnormality seen in Caucasian azoospermic men with Sertoli cell-only syndrome. AZF deletions were detected in 9% of men with a normal karyotype and for whom DNA was available for analysis. Currently, AZF deletions are routinely examined in an infertile population with nonobstructive azoospermia or severe oligozoospermia. However, ~10 years ago, AZF deletions were not tested in every patient, explaining the difference in numbers of patients tested for karyotype analysis and Yq microdeletions.

In the Center for Reproductive Medicine, currently 3.10% of all ICSI cycles performed include male patients with NOA (after a successful TESE). Another 2.24% of patients have an obstructive azoospermia. Overall, the number of ICSI cycles performed with sperm cells from azoospermic male patients is very low (~5%).

In a recent study, Vloeberghs et al. [9] looked at the success rate of TESE ICSI cycles in patients with NOA. From the 714 patients included in the study, 464 were diagnosed as having “SCOS.” For 38.4% of these patients, mature sperm cells could be detected. Consequently, for the majority of these patients, no spermatozoa could be retrieved. Since this study and our study are (partly) retrospective studies, we were not able to look for the presence of spermatogonia by testing molecular markers. Consequently, the classification of SCOS patients in “complete” or “incomplete” SCOS is only based on the data obtained from (multiple) TESE attempts and a biopsy sample for diagnostics purposes.

For the final selection of patients for the present study, only Caucasians were included with an idiopathic Sertoli cell-only syndrome and for whom no sperm cells were found in multiple ejaculate samples, a diagnostic testis biopsy and, if available, a therapeutic TESE sample. Nine patients who fulfilled all criteria were included for further studies. The presence of a gr/gr deletion could be excluded, due to testing in previous studies [11].

These patients were analyzed through array CGH in order to detect CNVs that might be related to their fertility problems. Previous studies, performed in our center or abroad, have already shown that some CNVs might be related to male infertility. In the study of Krausz et al. X-linked CNVs were more abundant in the infertile patient group [12]. In our study, one X-linked CNV was detected that was absent in the control group. This CNV is removing multiple copies of the Cancer-Testis 45 gene family. In humans, at least six members of this family are present. The CT45 gene is expressed in testicular tissues, as well as in different cancer tissues. However, the role of this gene family in spermatogenesis remains unknown. We have not verified the presence of copy number variations in this gene since no Taqman copy number assays were available for this region. Furthermore, multiple copy number variations have been reported in the database of genomic variants (http://dgv.tcag.ca/dgv/app/home). When considering all X-linked CNVs detected in this study, three CNVs could be identified in the patient group and 15 in the control group, indicating that 0.3 and 0.7 X-linked CNVs were, respectively, detected per patient or per control. These X-linked patient CNVs were the one reported above involving the CT45 gene family and another CNV affecting TEX28 in two patients. In 5/23 controls, this region was also duplicated/deleted (and consequently the frequencies were similar). However, it should be noted that the average spacing of the probes on the X-chromosome specific array used in the study of Krausz et al. is much higher than the average resolution of the array of this study (4 kb versus 8.9 kb) [12].

We did detect, however, 30 patient-specific CNVs in which one or more genes were located. From these regions, five were selected out for further analysis. On the one hand, qPCR analysis was performed to confirm the presence of the CNV and to be able to test more control samples. On the other hand, the expression pattern of the genes located in these CNV regions was tested where appropriate. The knowledge of the expression pattern is useful in understanding the necessity of this gene in spermatogenesis. Genes that are not expressed in testicular tissues are not/less likely to be involved in spermatogenesis. Through qPCR analysis, we could conclude that the region 16p13.11 is very polymorphic, and consequently the PDXDC1 gene located in this region is presumably not crucial for spermatogenesis. Also the region containing C18ORF26 is most likely not involved in spermatogenesis since no transcripts of this gene were detected in testicular tissues. In a recent study, we have sequenced all (mRNA) transcripts from testicular tissues (unpublished data). Here again, we could show that C18ORF26 is not expressed in testicular tissues, while all other analyzed transcripts (including MTHFD2L) were present. We currently have no explanation for the discrepant results for the MTHFD2L gene. Massive parallel sequencing of RNA from testicular tissues from multiple patients, analyzed each individually, was able to show the presence of this gene, while PCR amplification with multiple primer sets from different commercially available testis libraries and in-house isolated testicular mRNA failed to amplify fragments of this gene.

The remaining two regions contained at least one gene that was shown or known to be expressed in testicular tissues: PRCC was tested in our center, while the expression pattern of ZMYM5 was described by Sohal et al. [13].

We also checked the presence of noncoding RNA sequences in regions where no known genes were located. Several testicular RNA transcripts were detected, but since their function and importance remain unknown, we decided not to focus on these regions for this paper.

Overall, we can conclude from this study that multiple CNVs could be (partially) causal for SCOS. However, it remains hard to determine the functional importance of genes located in these regions. In the present study ~24 CNVs were detected per individual (patient or control) analyzed. Part of the CNVs were unique for the control group. These neutral (or at least not-fertility causing) CNVs might be rare polymorphisms. Similarly, also the CNVs detected in the patient group might be rare CNVs, not related to the fertility problems of the patients. Consequently, array CGH might be useful in a research setting investigating male infertility, but it is still too early to implement as a routine test for idiopathic cases.

Altogether, these data show that especially the sex chromosomes are involved in the etiology of Sertoli cell-only syndrome, which was already suggested >20 years ago [14]. Moreover, Krausz et al. showed that CNVs on the X chromosome might be involved in male infertility [12]. Currently, the most frequent cause of nonobstructive azoospermia with Sertoli cell-only syndrome as the phenotypic background remains Klinefelter syndrome. Given the availability of next generation sequencing technologies, allowing genome sequencing as well as a better characterization of transcripts involved in spermatogenesis, it is expected that the knowledge of genetic factors involved in the etiology of Sertoli cell-only syndrome will increase over the next few years.

## Supplementary Material

The supplementary table gives the Taqman Copy Number Assays used. The table gives the chromosomal region and the genomic position of the assay as well as its position according to the potential gene of intrest located in the CNV region. More information is available online (http://www.thermofisher.com).

## Figures and Tables

**Table 1:** Overview of patient-specific copy number variations and the genes located in these regions, detected by array comparative genomic hybridization analysis.

	Patient	Deletion/ duplication	Region	Start position	Stop position		Genes involved
1	ESCO16	Del	1p21.1	104067184	104321250	13	RNPC3/AMY2B/AMY2A/AMY1A/AMY1C/AMY1B
2	**ESCO2**	**Dupl**	**1q23.1**	**156764562**	**156796736**	**7**	**PRCC/SH2D2A/NTRK1**
3	ESCO29	Dupl	2p22.1	38956776	38968369	3	GALM
4	ESCO2	Del	2p15	62258290	62277149	4	COMMD1
5	ESCO36	Del	2q13	110833640	110983703	20	MALL/NPHP1
6	**ESCO19**	**Del**	**4q13.3**	**75019400**	**75117648**	**10**	**MTHFD2L**
7	ESCO19	Dupl	4q21.1	76504370	76521476	2	CDKL2
8	ESCO34	Del	5p14.3	21943466	22002677	6	CDH12
9	ESCO25	Del	5p13.1	40779232	40785703	2	PRKAA1
10	ESCO29	Del	6p12.1	52624027	52682610	9	GSTA2/GSTA1
11	ESCO28	Del	6q26	162618199	162825032	23	PARK2
12	ESCO25	Dupl	7p22.2	3144476	3458017	28	SDK1
13	ESCO19	Del	7q11.22	66671745	66672634	2	TYW1
14	ESCO29	Del	7q35	143884029	143953472	4	FLJ43692/OR2A42/OR2A1
15	ESCO2	Dupl	8p23.1	6828426	6837339	2	DEFA1
16	ESCO34	Del	8p22	15403439	15409232	2	TUSC3
17	ESCO34	Dupl	11q24.2	124743538	124798029	11	ROBO3/ROBO4/HEPN1/HEPACAM
18	ESCO36	Del	12p12.2	21011077	21404166	37	SLCO1B3/LST-3TM12/SLCO1B1
19	ESCO25	Dupl	12q14.2	63947732	64116568	6	DPY19L2
20	ESCO19	Del	12q21.32	86695679	86703030	2	MGAT4C
21	ESCO29	Del	12q23.1	99994977	100005332	2	ANKS1B
22	**ESCO29**	**Dupl**	**13q12.11**	**20419333**	**20445320**	**5**	**ZMYM5**
23	ESCO36	Del	15q14	34671574	34841446	17	GOLGA8A/GOLGA8B + MIR1233-/DQ593032/DQ582939/KI110855
24	**ESCO18**	**Del**	**16p13.11**	**14968855**	**15115579**	**8**	**NOMO1/NPIP/PDXDC1**
25	ESCO28	Dup	16q12.2	55832511	55865159	5	CES1
26	**ESCO2**	**Del**	**18q21.2**	**52212057**	**52306572**	**10**	**C18orf26**
27	ESCO25	Del	18q21.31	55931229	55936547	2	NEDD4L
28	ESCO36	Dupl	22q11.21	18894835	19010508	19	DGCR6/PRODH
29	ESCO25	Del	22q13.33	50296855	50301369	2	ALG12
30	ESCO16	Dupl	X	134778328	134910134	12	CT45-1/CT45-2/CT45-4/CT45-3

**Table 2:** Overview of patient-specific copy number variations not containing any genes.

Chromosome	Start	End	
12p12.1	21565934	21580503	—
16q23.3	83912597	83920609	—
2q14.3	122828556	122935716	DQ591124 (piRNA) and DQ583822 (piRNA)
3q13.32	118225639	118247407	EU250752
4p14	38458265	38467938	—
4p15.1	34033992	34053693	BC036345
5p14.3	20599622	20712049	AK093362
5p15.2	12559564	12572720	—
5q23.2	127077075	127082714	—
6p22.3	19774967	19791138	—
7q21.12	86941358	86947442	—
8q11.22	52042609	52074026	—
8q21.3	90423856	90458375	—
9p23	12163230	12357073	DB098556, HY017233, HY200407, and DB448686
