# Supplementary material for: Sertoli Cell-Only Syndrome: Behind the Genetic Scenes
Source: Biomed Res Int. 2016 Jan 26;2016:6191307. doi: 10.1155/2016/6191307 (PMC4746273; doi:10.1155/2016/6191307)
Supplement: Supplementary file 1 — The supplementary table gives the Taqman Copy Number Assays used. The table gives the chromosomal region and the genomic position of the assay as well as its position according to the potential gene of intrest located in the CNV region. More information is available online (http://www.thermofisher.com). [file 6191307.f1.pdf]

Region	Assay	Gene	Location on Transcript or (	Location chromosom hg19
**1q23.1**	Hs02369977_cn	PRCC	Overlaps Exon 7	156770617
**4q13.3**	Hs02394202_cn	MTHFD2L	Overlaps Exon 3 - Intron 3	75041091
**13q12.11**	Hs02812305_cn	ZMYM5	Within Exon 3	20426264
**16p13.11**	Hs03938043_cn	PDXDC1	Within Intron 1	15073771
**18q21.2**	Hs02394202_cn	C18ORF26	Overlaps Exon 2 - Intron 2	52262343

Supplementary table: overview of the Taqman copy number assays
